# A Concerted Action Of Estradiol And Insulin Like Growth Factor I Underlies Sex Differences In Mood Regulation By Exercise

**DOI:** 10.1038/srep25969

**Published:** 2016-05-12

**Authors:** Victor Munive, Andrea Santi, Ignacio Torres-Aleman

**Affiliations:** 1 Cajal Institute, CSIC, Madrid, Spain; 2 CIBERNED, Madrid, Spain

## Abstract

Mood homeostasis present sexually dimorphic traits which may explain sex differences in the incidence of mood disorders. We explored whether diverse behavioral-setting components of mood may be differentially regulated in males and females by exercise, a known modulator of mood. We found that exercise decreases anxiety only in males. Conversely, exercise enhanced resilience to stress and physical arousal, two other important components of mood, only in females. Because exercise increases brain input of circulating insulin-like growth factor I (IGF-I), a potent modulator of mood, we explored whether sex-specific actions of exercise on mood homeostasis relate to changes in brain IGF-I input. We found that exercise increased hippocampal IGF-I levels only in cycling females. Underlying mechanism involved activation of estrogen (E_2_) receptors in brain vessels that led to increased uptake of serum IGF-I as E_2_ was found to stimulate IGF-I uptake in brain endothelial cells. Indeed, modulatory effects of exercise on mood were absent in female mice with low serum IGF-I levels or after either ovariectomy or administration of an E_2_ receptor antagonist. These results suggest that sex-specific brain IGF-I responses to physiological stimuli such as exercise contribute to dimorphic mood homeostasis that may explain sex differences in affective disorders.

There is a recognized sex bias in mood disorders with sex-specific incidence rates in several of them^1^. While ongoing research is improving our understanding of sex variance in brain function (see for example ref. 2 for a recent review), the fact is that until relatively recently, sex-specific traits have been a widely neglected area in brain studies. Fortunately, these variances are now increasingly recognized as an important determinant of brain physiology and disease^3^. Conceivably, underlying differences in sex incidence of mood disorders may include distinct mechanisms of mood homeostasis as evidenced for example by the modulatory action of the ovarian hormone estradiol on female anxiety^4^. Other sex-specific mechanism may involve insulin-like growth factor I (IGF-I), a pleiotropic neuroroprotective signal^5^ with potent mood regulatory actions^6^,^7^,^8^,^9^. For example, oscillations in brain IGF-I levels along the rat estrous cycle have been reported^10^,^11^, and sex-dependent differences have been documented in response to changes in brain IGF-I signaling^12^,^13^. Thus, sex differences in brain IGF-I function are starting to be documented.

Exercise is a known modulator of mood and arousal, two mutually influencing behavioral components affecting cognition^14^,^15^,^16^,^17^,^18^. Exercise is starting to be used as a therapeutic maneuver in different brain diseases^19^,^20^. Interestingly, the beneficial effects of exercise on mild cognitive impairment have been seen to be sexually dimorphic^21^. In turn, exercise and other arousing stimuli such as environmental enrichment elicit the entrance of serum IGF- I into the brain^22^,^23^. Importantly, exercise and environmental enrichment are neuroprotective in part through serum IGF-I^24^,^25^.

The hippocampus participates in brain responses to exercise even though it is not directly involved in motor control^26^. In this study we focused on the hippocampus as a brain region particularly relevant to mood disorders^27^, that is linked to major brain areas related to anxiety^28^, and actively captures serum IGF-I in response to exercise^22^. These characteristics make the hippocampus an adequate area to analyze possible connections between IGF-I and mood modulation through exercise. As various types of exercise regimes elicit sex-dependent changes in serum IGF-I levels in different species, including humans^29^, we explored whether capture of serum IGF-I by the brain in response to exercise is sexually dimorphic and relates to sex differences in mood homeostasis.

## Results

**Figure 1:**
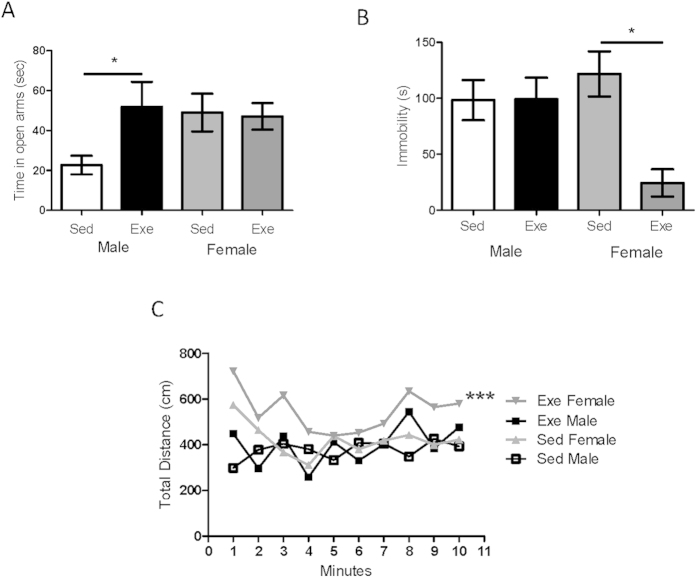
Sex differences in mood modulation by exercise. (**A**) Sedentary female mice show lower anxiety in the elevated plus maze as they spent significantly more time in the open arms (n = 10 per group; ^#^p < 0.05 vs males). However, in response to exercise male mice spent significantly more time in the open arm of the elevated plus maze while female mice did not show any change (*p < 0.05 vs sedentary males; n = 8 per group). (**B**) Resilience to stress is increased by exercise only in female mice. Exercised female mice spent more time active in the tail suspension test. (n = 5 per group; p < 0.05 vs respective control). (**C**) Physical arousal is increased in female mice by exercise, but not in males. Vestibular stimulation elicited increased locomotor activity only in exercised females (***p < 0.001 vs sedentary females, n = 10 for all groups).

Anxiety levels, -measured in the elevated plus maze as time spent in the open arms, were significantly lower in female mice as compared to males (Fig. 1A), which agrees with previous observations^30^. These differences in emotional tone may reflect sex differences in mood homeostasis. For example, ovarian hormones modulate anxiety^4^,^31^. Indeed, we observed a significant increase in anxiety levels after ovariectomy (see Supplementary Figure 1A). Other sex differences such as higher levels of miR375 in males, that are directly related to stress^32^, may also contribute to differing mood homeostasis (see Supplementary Figure 1B).

We set out to investigate further possible differences in mood homeostasis between sexes by examining the effect of physical activity, a physiological modulator of diverse components of mood^33^. We found that treadmill running reduced anxiety in male mice while females remained unaffected (Fig. 1A). This may be related to relatively low baseline anxiety seen in females as compared to males (Fig. 1A). Conversely, when mood homeostasis was disturbed by exposure to stress (forced swimming), exercise increased resilience to stress, a behavioral state reflecting mood, only in females. Thus, re-exposure after exercise to a stress challenge such as the tail suspension test resulted in greater mobility time in female mice, indicating better coping to behavioral despair (Fig. 1B). Greater mobility in exercised females was not due to a female-specific generalized increase of activity, as both sexes exhibited similar activity after exercise (not shown). Stress resilience has also been found to be sexually dimorphic after environmental enrichment^34^. To explore further sex differences we measured physical arousal after vestibular stimulation, a behavioral-setting component of mood that is estrogen-dependent^35^. We found that exercise enhanced arousal only in females, as they showed increased mobility after vestibular stimulation (Fig. 1C). In accordance with previous observations on the lack of effects of estrogens on other types of arousal stimuli^35^, neither olfactory nor somatosensory stimulation elicited significantly different responses between males and females and exercise did not affect them either (not shown), confirming again that exercise does not increase mobility only in females.

### Sex differences in exercise modulation of hippocampal IGF-I

**Figure 2:**
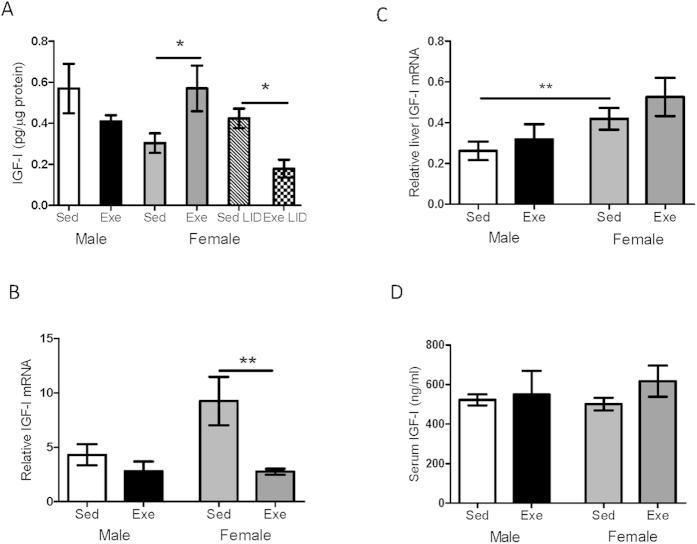
Sex differences in hippocampal IGF-I responses to exercise. (**A**) In female mice, exercise increased hippocampal levels of IGF-I. However, female mice with low serum IGF-I levels (LID) showed reduced hippocampal IGF-I after exercise (n = 10 per group; *p < 0.05). (**B**) Exercise reduced IGF-I mRNA in wild type female mice (n = 10 per group; **p < 0.01 vs sedentary females). (**C**) While female mice have higher liver IGF-I mRNA levels than males, exercise slightly increased them in both sexes (n = 8 per group; **p < 0.01 vs sedentary males). (**D**) Exercise did not modify similar basal serum levels of IGF-I in male or female mice (n = 10 per group).

Because in male mice anxiolytic actions of exercise depend in part on serum IGF-I^36^, we examined whether exercise elicits sex dimorphic responses in the entrance of this circulating growth factor into the brain. We found that exercise (treadmill running) significantly increased hippocampal IGF-I levels only in female mice (Fig. 2A). Because previous observations showed that increased brain IGF-I levels after exercise are mediated by its uptake from the circulation^22^, we examined the effect of exercise in mutant female mice with low circulating IGF-I (LID mice). Female LID mice did not show increased hippocampal IGF-I levels after exercise; rather the opposite, IGF-I was decreased by exercise (Fig. 2A). In agreement with an external source of IGF-I explaining the increase after exercise in normal mice we observed that hippocampal IGF-I mRNA was decreased after exercise only in females (Fig. 2B).

As the cerebellum is actively involved in motor coordination we analyzed whether exercise modulated IGF-I levels in this brain region and found no changes (see Supplementary Figure 1C). We also determined whether a bout of exercise modifies IGF-I levels in the hippocampus and found a non significant trend to increased levels in male mice (see Supplementary Figure 1D), as already reported^22^. Thus, sex-specific responses to exercise are exercise-regime dependent and brain region-specific. No differences in IGF-I and insulin receptors in hippocampus were observed between males and females in response to exercise (not shown), indicating that sex-specific changes of these receptors cannot explain the observed changes in hippocampal IGF-I.

The observed mismatch between high IGF-I levels and low IGF-I mRNA in exercising females agrees with the notion that increases in hippocampal IGF-I originate from its enhanced uptake from serum. As serum IGF-I levels may increase in response to specific types of exercise^29^, and the liver is the main producer of circulating IGF-I^37^ we checked whether liver IGF-I mRNA responds to exercise in a sex-specific pattern. While female mice had higher levels of liver IGF-I mRNA than males under sedentary conditions, both sexes show a similar non significant increase in IGF-I mRNA (Fig. 2C). Differences in basal liver expression of IGF-I mRNA between males and females were not reflected in serum levels; both sexes had similar levels that were not changed by exercise (Fig. 2D). Thus, increased brain uptake of serum IGF-I is not a consequence of increased serum IGF-I after exercise.

### Role of 17β estradiol in female responses to exercise

**Figure 3:**
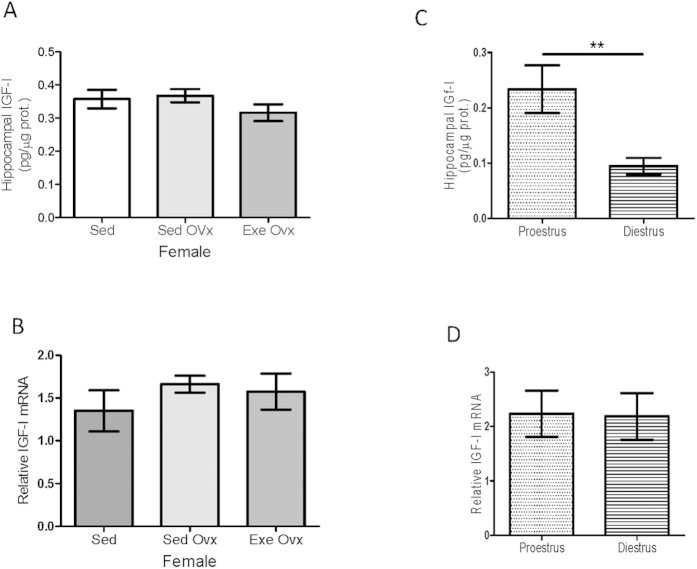
Ovarian modulation of hippocampal IGF-I. (**A**) In ovariectomized female mice exercise did not increase IGF-I levels in hippocampus (n = 8). (**B**) Similarly, exercise did not modify hippocampal IGF-I mRNA levels (n = 8 per group). (**C,D**) Levels of hippocampal IGF-I (**C**), but not of its mRNA (**D**) vary along the estrous cycle (n = 6 per group; *p < 0.05 vs proestrus).

We next determined whether estradiol (E_2_) participates in female-specific responses of hippocampal IGF-I to exercise as these two hormones are known to interact with each other^38^,^39^. First, we found that ovariectomy abrogated changes in IGF-I and its mRNA in response to exercise (Fig. 3A,B). We also observed that hippocampal IGF-I is higher at proestrus, when E_2_ levels are highest, while IGF-I mRNA does not change along the estrous cycle (Fig. 3C,D). Supporting the notion that changes in hippocampal IGF-I along the estrous cycle rely on differential uptake from the circulation we did not observe any change in hippocampal IGF-I in LID females along the estrous cycle (not shown). The fact that after ovariectomy sedentary females did not show reduced hippocampal IGF-I, as seen during diestrus, probably reflects that changes in brain neuroactive steroids after gonadectomy do not reflect changes in the circulation^40^. In addition, diestrus produces a short-term reduction of E_2_ whereas one month after ovariectomy reduced levels of E_2_ are constant and compensatory mechanisms may be operant.

**Figure 4:**
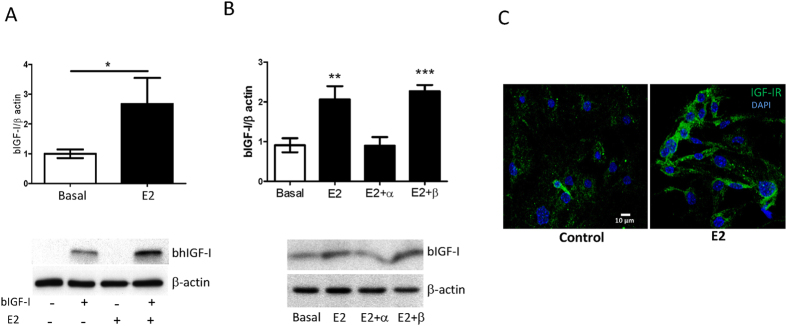
Estradiol stimulates uptake of IGF-I by brain endothelial cells. **(A**) Cultured brain endothelial cells accumulate significantly more IGF-I after treatment with 10^−10^M estradiol (E_2_). Representative blot is shown (*p < 0.05 vs control; n = 8 per group). (**B**) The effects of E_2_ were mediated by α E_2_ receptors as only the α inhibitor MPP blocked E_2_ actions. Representative blots are shown (*p < 0.05; n = 8). (**C**) Levels of IGF-I receptor (green) at the cell membrane of endothelial cells are markedly increased three hours after addition of 10^−10^M E_2_.

To test whether E_2_ influences hippocampal IGF-I levels by modulating uptake of IGF-I from the circulation we examined the role of this sex hormone on IGF-I uptake by brain endothelial cells. We found that E_2_ increased uptake of IGF-I by cultured brain endothelial cells (Fig. 4A), through the α estrogen receptor (Fig. 4B). In addition, treatment of endothelial cells with E_2_ promoted higher levels of IGF-I receptor at the cell membrane (Fig. 4C). In turn, IGF-I uptake by choroid plexus epithelium was not affected, indicating site-specific actions of E_2_ (see Supplementary Figure 2A). The latter also indicates that E_2_ does not produce a generalized increased in blood-brain-barrier (BBB) permeability as both cell types form part of the BBB and mediate the transfer of IGF-I to the brain^23^,^41^. Importantly, when the effects of E_2_ were analyzed in endothelial cells obtained from male or female mice, in both cases E_2_ was able to stimulate IGF-I uptake (see Supplementary Figure 2B). This suggests that differential uptake of IGF-I is related to E_2_ rather than to sex-specific differences in endothelial function.

**Figure 5:**
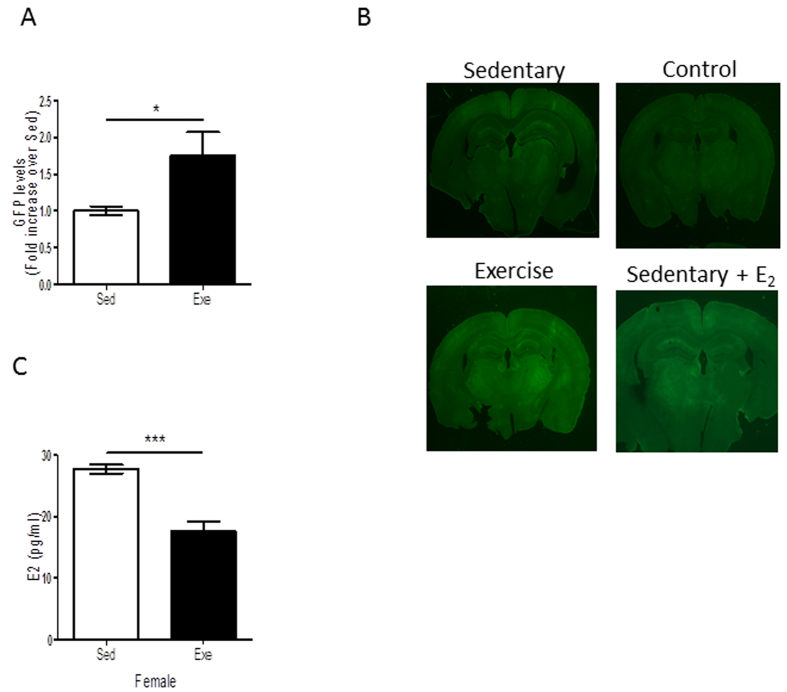
Exercise stimulates estrogen receptors in brain vessels. (**A**) Levels of GFP were significantly increased in the brains of exercised female mice expressing an estrogen receptor GFP reporter (*p < 0.05 vs sedentary females; n = 3 per group). (**B**) Exercise activates estrogen receptor GFP reporter activity (expressed by brain vessels, see Supplementary Figure 3A), as reflected by increased GFP staining. Representative brain slices of non-injected female mice (control), injected with an E_2_ receptor-GFP reporter and exercised (exercise), non-exercised (sedentary), and non-exercised but injected with E_2_ (sedentary + E2). (**C**) Exercise decreased serum E_2_ levels in female mice.

We then explored the effects of E_2_ on brain endothelial cells during exercise. We injected female mice in the carotid artery with a virus expressing a GFP reporter of the estrogen receptor^42^, allowing us to determine estrogen receptor activity in endothelium (see Supplementary Figure 3). We found that exercise elicited higher levels of GFP in exercised females (Fig. 5A,B). However, exercise reduced serum E_2_ in female mice (Fig. 5C). Hence, increased E_2_ receptor activity in brain endothelium in response to exercise is not reflecting increased serum E_2_ levels. Together with the blockade of exercise-induced increases in hippocampal IGF-I after ovariectomy (Fig. 3A), these data suggest that increased activity of E_2_ receptors in brain endothelium in response to exercise translates into a greater uptake of IGF-I by these cells and explains higher levels of IGF-I in the female hippocampus after exercise.

**Figure 6:**
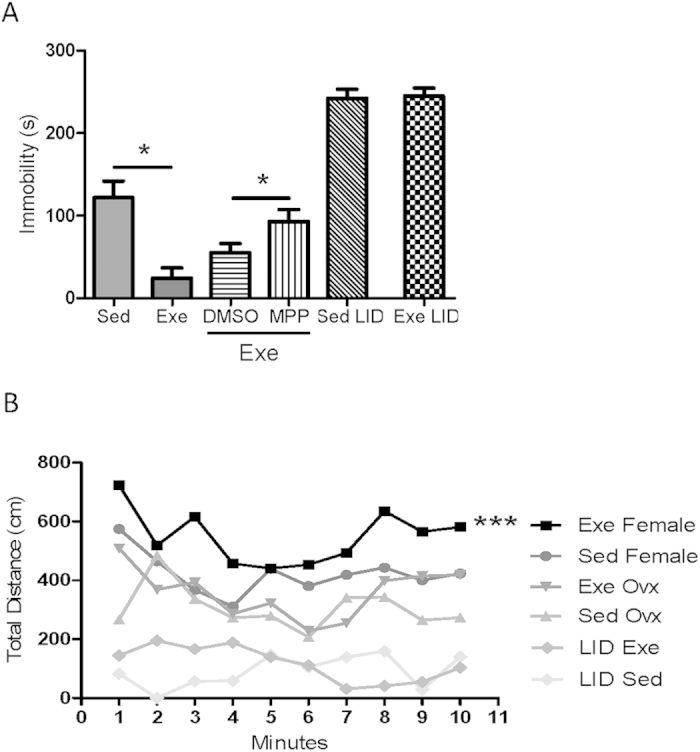
Estradiol and IGF-I mediate actions of exercise on female mood. (**A**) Exercise modulates resilience to stress as measured in the tail suspension test in an estrogen- and serum IGF-I-dependent manner. Administration of the estrogen receptor α antagonist MPP during exercise resulted in significantly attenuated mobility. DMSO group is the vehicle group of the MPP treatment. Similarly, female mice with low serum IGF-I levels (LID mice) did not show improved resilience to stress after exercise (*p < 0.05; n = 5–10 per group). (**B**) Exercised LID female mice or ovariectomized wild type female mice did not show increased motor activity after vestibular stimulation (***p < 0.001 vs sedentary; n = 10 each group). Both groups of animals showed reduced basal motor activity.

To confirm a role of E_2_ and IGF-I in mood modulation by exercise we determined resilience to stress in LID females and in wild type females administered an E_2_ receptor α antagonist (MPP) during exercise. We found that in both cases increased resilience to stress after exercise was significantly attenuated (Fig. 6A). Moreover, both E_2_ and IGF-I seem necessary for exercise to enhance physical arousal as either ovariectomized wild type or intact LID female mice did not show changes in arousal after exercise. As a matter of fact, both ovariectomized and LID females showed lower basal levels of arousal than wild type sedentary females (Fig. 6B), suggesting that basal levels of arousal are also regulated by E_2_ and IGF-I.

## Discussion

These results indicate that sex differences in mood homeostasis are reflected in sex-dependent effects of exercise on mood modulators such as emotional tone and physical arousal. Estrogen and IGF-I -two hormones that are well known to interact with each other^38^,^39^ and to modulate mood, act in concert in the observed female-specific differences. Thus, estrogen is involved in the modulatory actions of exercise in females, and at the same time promotes brain uptake of serum IGF-I through the BBB. In turn, IGF-I is also required for female-specific mood responses to exercise.

It is interesting to note that anxiolytic actions of E_2_ have been usually described to be mediated through the β E_2_ receptor^43^,^44^,^45^, while in our case it is the α receptor the one that is mediating E_2_ effects. Conceivably, E_2_ modulates anxiety through its two types of receptors acting at different pathways and/or brain sites. These possibilities merit further study. Also, whether increased uptake of serum IGF-I is necessary for estrogen to influence exercise modulation of mood requires further analysis. However, the fact that serum IGF-I is required for exercise actions on mood favors the possibility that E_2_ upregulation of IGF-I uptake is a necessary step. In turn, the role of other ovarian hormones such as progesterone, with established mood regulatory effects^31^,^46^ needs further perusal as ovariectomy abolished the mood modulatory actions of exercise.

Previous observations already indicated that estradiol differentially modulates BBB function in males and females^47^. In males, this E_2_/IGF-I pathway is not present and exercise modulates mood by improving emotional tone through as yet unknown mechanisms. The latter agrees with previous findings of IGF-I-independent anxiolytic actions of exercise in male mice^36^,^48^. It is possible that regulation of brain IGF-I levels by sex steroids may also relate to the role of IGF-I as a central modulator of the reproductive axis^49^, thereby establishing a feedback loop; however, this possibility remains to be studied.

These findings indicate that stress, arousal, and anxiety, with intertwined influences among them^2^,^50^,^51^, are distinctly regulated in males and females, highlighting the existence of different regulatory layers in mood homeostasis. Thus, exercise modulates mood in both sexes acting through different mechanisms addressing distinct components of mood. These data allow us to speculate that decaying ovarian function along aging may contribute to reduced serum IGF-I input to the brain and altered mood homeostasis known to be associated to the aging process. While in aging male mice brain IGF-I levels are reduced^52^, we need further studies to determine whether female old mice also have decreased brain IGF-I levels and how they compare to male levels.

Although with a different pattern to that seen in rats^10^, mice show sexual dimorphism in hippocampal levels of IGF-I. Basal differences between males and females were in part independent of ovarian hormones, while those seen in response to exercise fully depended on intact ovarian function. Taken together, it appears that sex differences in hippocampal IGF-I are established during the so called “organizational period” taking place early during the sexual differentiation of the brain, whereas regulation by exercise of the adult sex-dependent pattern is sensitive to ovarian steroids such as E_2_. Because exercise is becoming a popular non-pharmacological tool for treatment of mood disorders^53^, the observed male/female differences in the effects of exercise in rodents suggests that therapeutic interventions should take into account potential sex differences in humans.

In summary, sex-specific responses to physiological neuroprotective stimuli such as physical activity, that modulate mood in part through modulation of endocrine signals, contribute to sex differences in mood homeostasis. Hopefully, a better understanding of these differences will help us gain insight of sex differences in the incidence of mood disorders.

## Methods

### Animals and experimental procedures

Female and male adult C57BL/6J mice (19–23 g 8–9 wk old; Harlan Laboratories, Spain) and female liver-specific IGF-I-deficient mice (LID) were housed in standard cages (48 × 26 cm^2^) with 5 animals per cage. Mice were kept in a room with controlled temperature (22 °C) under a 12-12 h light-dark cycle; fed with a pellet rodent diet and water ad libitum. All experimental protocols were performed during the light cycle. Animal procedures followed European guidelines (86**/**609**/**EEC and 2003**/**65**/**EC, European Council Directives) and were approved by the local Bioethics Committee (Madrid Community Government). Estrous cycle in female mice was monitor by daily inspection of vaginal smears. The material was collected at the same time each day during 10 days. Approximately 10 μl of 0.9% saline were gently flushed into the vagina with the tip of a plastic pipette three times, and the final flush placed onto a glass slide and observed under the light microscope with a 10 × objective. The determination of the estrous cycle phase was based on the proportion among these cell types: predominance of leukocytes (Diestrous), predominance of nucleated epithelial cells (Proestrous), predominance of cornified epithelial cells (Estrous), and a mix of cell types with a predominance of leukocytes and a few nucleated epithelial and/or cornified squamous epithelial cells (Metestrus). The E_2_ receptor α antagonist (MPP) was administrated by intraperitoneal injection at doses of 10 μg/ml in DMSO. Treatment started one day before treadmill training, and continued during all the training period. Daily injections were given after running to avoid interference with exercise performance. Controls receive equivalent DMSO injections.

### Treadmill running

Mice were subjected to treadmill running for 2 weeks (5 days/week). Mice were familiarized with the treadmill apparatus (Letica, Italy) to minimize novelty stress and then divided in two groups: exercised and non-exercised. The electrical shock system that encourages the animals to run was disconnected to avoid pain stress. The exercise group ran for 40 min at 12 m/min, whereas the control group remained for the same time in the treadmill without running. We chose this mild intensity exercise regime to avoid changes in stress hormones^54^ that could interfere with post-exercise behavioral assessment. For biochemical assays, a subset of mice were deeply anesthetized and sacrificed right after the last running session. Trunk blood samples were obtained, and brains were perfused with 0.09% saline solution and snap frozen for ELISA, Western blot and qPCR or were further perfused with 4% paraformaldehyde for immunostaining. Additional groups of animals were used for behavioral testing at different times after running (see below).

### Surgery

#### Ovariectomy

Mice were anesthetized by intraperitoneal injection of a mixture of ketamine (50 mg/kg) and xylazine (4.5 mg/ml). The ovaries were isolated by ligation of the oviduct before removal. Sham mice were subjected to the same procedure without removing the ovaries.

#### Intracarotid virus injection

Mice were anesthetized with isoflurane (with oxygen flux at 0.8–1 l/min). The common carotid artery was exposed, the external carotid ligated and 50 μl of virus suspension (4.3 × 10^10^ pfu/ml) injected using a 100 μl Hamilton syringe. A guide cannula was inserted in the common carotid artery. After surgery, the mice displayed normal food intake and water consumption as well as spontaneous locomotion. Two weeks later, after fully recovered, they were submitted to treadmill running.

### Elevated Plus Maze

A five minutes elevated plus maze (EPM) test was performed as described^55^, with sedentary and exercised groups, one day after the last running session. Tracking was carried out with Video Tracking Plus Maze Mouse (Med Associates, USA)

### Stress Resilience

Three days before treadmill training mice were exposed once to forced swim as a stressor. One day after completion of exercise training, the tail suspension test (TST) was used to measure coping behavior^56^. We used this second type of stressful test to avoid re-exposure of mice to the first stressor (forced swim) and in this way eliminate any potentially confounding learning component. In the forced swim test (FST) mice were placed in a glass cylinder (12 cm diameter, 29 cm height) filled with water (23 °C) to a height of 15 cm, the duration of the test was 6 min, and scored the last 4 minutes, by a blind observer. In the TST, mice were individually suspended by the tail from a plastic cage (21 × 26 × 15) using adhesive tape (distance from tip of tail was 2 cm), the distance from the floor was 35 cm. A 6 min test session was videotaped and time spent immobile was scored by a blind observer.

### Arousal Assay

Mice were exposed to a series of three sensory stimuli as described^57^, in the following order: olfactory, tactile and vestibular. Baseline values were obtained one day before treadmill training. Post-exercise arousal tests were initiated 30 min after finishing the last running session. Arousal stimuli were given with a 15± 5 min interval. Behavioral responses were measured by recording cage activity in either the horizontal or vertical direction. Data were collected onto a PC using Versamax Software (Accuscan Instruments, USA)

### Immunoassays

Western blot (WB) was performed as described^58^. IGF-I in serum and tissues was determined using a species-specific ELISA (R&D Systems, USA), as described^59^. Blood was collected from the heart after pentobarbital anesthesia before trans-cardiac perfusion.

### Cell cultures and *in vitro* assays

Endothelial and choroid plexus cell cultures were performed as described^60^, with minor modifications. Cells were washed with PBS and serum-free DMEM/F12 without red phenol was added. Three hours later, 1 nM 17β-estradiol (Sigma, USA) was added (or equivalent dose of DMSO in control wells) and 2 hours later biotinylated IGF-I (bIGF-I, IbmH, Germany, 0.2 μg/ml). One hour later cells were lysed and processed for WB. For analysis of surface IGF-I receptors, endothelial cells were plated one day prior to staining in order to achieve 60–80% confluency. Cells were washed with PBS and fasted for three hours with serum-free DMEM/F12 without phenol red. Subsequently, 1 nM 17β-estradiol or equivalent dose of DMSO was added. Two hours later, cells were blocked with a 0.1% BSA /PBS at 4 °C for 5 minutes and washed with PBS 2 times (5 min/Wash). Cells were incubated with IGF-IRα conjugated antibody (Neuromics) at 1:20 dilution in 0.1% BSA /PBS in a humidified chamber for 30 min at 4 °C. Then, cells were washed 3 times in 0.1% BSA/PBS (5 min/wash) protected from light at 4 °C. Finally, cells were fixed with 4% paraformaldehyde for 10 minutes at room temperature, counterstained with Hoetchst at 1:500 in 0.1% BSA/PBS and washed with PBS.

### qPCR

mRNA was extracted with TRIZOL (Life Technologies, USA) following the manufacturer´s protocol using TaqMan probes. Reactions were performed in an ABI PRISM^®^ 7000 Sequence Detection System. mRNA levels were normalized with 18sRNA.

### Plasmid and viral constructs

The CMV promoter downstream of eGFP was substituted from the pLKO-eGFP vector by an ERE promoter (3X ERE TATA luc from AddGene, USA) to drive eGFP expression. For lentiviral vector production 80% confluent 293T cells were transfected for 4 h with 40 μg of a plasmid encoding the viral backbone (pHR’SIN), 10 μg of VSV-G envelope glycoprotein (pMDG.2) and 30 μg of packaging proteins (gag-pol, rev, tat, in pCMVΔR8.74) using 1 μM polyethyleneimine as a transfection agent. Cells were washed and refreshed with Dulbecco’s Modified Eagle Medium-DMEM containing glutamine, 10% Fetal Calf Serum and antibiotic mix. Viral particles were harvested after 36 h, filtered and stored frozen in 100 μl aliquots. For titration, ten-fold serial dilutions of the viral ERE-GFP construct (from undiluted to a dilution of 10^−7^) were done in DMEM Seed 6*10^4^ 293T Cells in each well of the 24-well cluster plate. After 72h cells were fixed with 4% paraformaldehyde for 30 minutes and immunostained for GFP.

### Statistical analysis

Statistical analysis was performed using GraphPad Prism 5 software (San Diego, CA, USA). All results are shown as mean ± s.e.m. For single comparisons, we used Student’s *t*-test and for multiple comparisons, one or two-way analysis of variance plus Bonferroni’s test. Probability values <0.05 were considered significant.

## Additional Information

**How to cite this article**: Munive, V. *et al.* A Concerted Action Of Estradiol And Insulin Like Growth Factor I Underlies Sex Differences In Mood Regulation By Exercise. *Sci. Rep.* **6**, 25969; doi: 10.1038/srep25969 (2016).

## Supplementary Material

Supplementary Information
