# Supplementary material for: A Concerted Action Of Estradiol And Insulin Like Growth Factor I Underlies Sex Differences In Mood Regulation By Exercise
Source: Sci Rep. 2016 May 12;6:25969. doi: 10.1038/srep25969 (PMC4864325; doi:10.1038/srep25969)
Supplement: Supplementary Information [file srep25969-s1.pdf]

Supplementary information

A CONCERTED ACTION OF ESTRADIOL AND INSULIN LIKE GROWTH FACTOR I UNDERLIE SEX DIFFERENCES IN MOOD REGULATION BY EXERCISE

Victor Munive, Andrea Santi, and Ignacio Torres-Aleman*

Cajal Institute, CSIC, and Ciberned. Madrid. Spain

SUPPLEMENTARY FIGURE LEGENDS

Supplementary Figure 1: A, Ovariectomized females (ovx) spent significantly less time in the open arms of the elevated plus maze than intact females (*p<0.05; n= 8 ovx and 10 intact). **B,** Hippocampal levels of miR375 are significantly higher in male mice (*p<0.05; n= 5 males and 4 females). **C,** Levels of IGF-I were not sexually dimorphic and did not change after exercise in the cerebellum (n=10 mice per group). **D,** An acute bout of treadmill running (1 hour) resulted in a slight, non-significant increase in hippocampal IGF-I levels in male mice (n=10 per group).

Supplementary Figure 2: A, Epithelial choroid plexus cells in culture accumulate biotinylated human IGF-I (bIGF-I), and estradiol ($E_{2,}$ 10^{-10}M) does not influence its uptake. Representative blot is shown (n=7). **B,** Cultured brain endothelial cells separately obtained from male and female mice show identical increased uptake of bIGF-I by E_2. Representative blot is shown (*p<0.05 vs basal levels; n= 8).

Supplementary Figure 3: A, Two weeks after intracarotid delivery of 50 µl of lentivirus expressing GFP, profuse vessel staining is observed (mouse cerebellum). **B,** C6 cells expressing ERE.GFP, a reporter construct of the E_2 receptor, show enhanced expression of GFP (green) in response to E_2 (10^{-10}M). Blue: DAPI staining of cell nuclei.

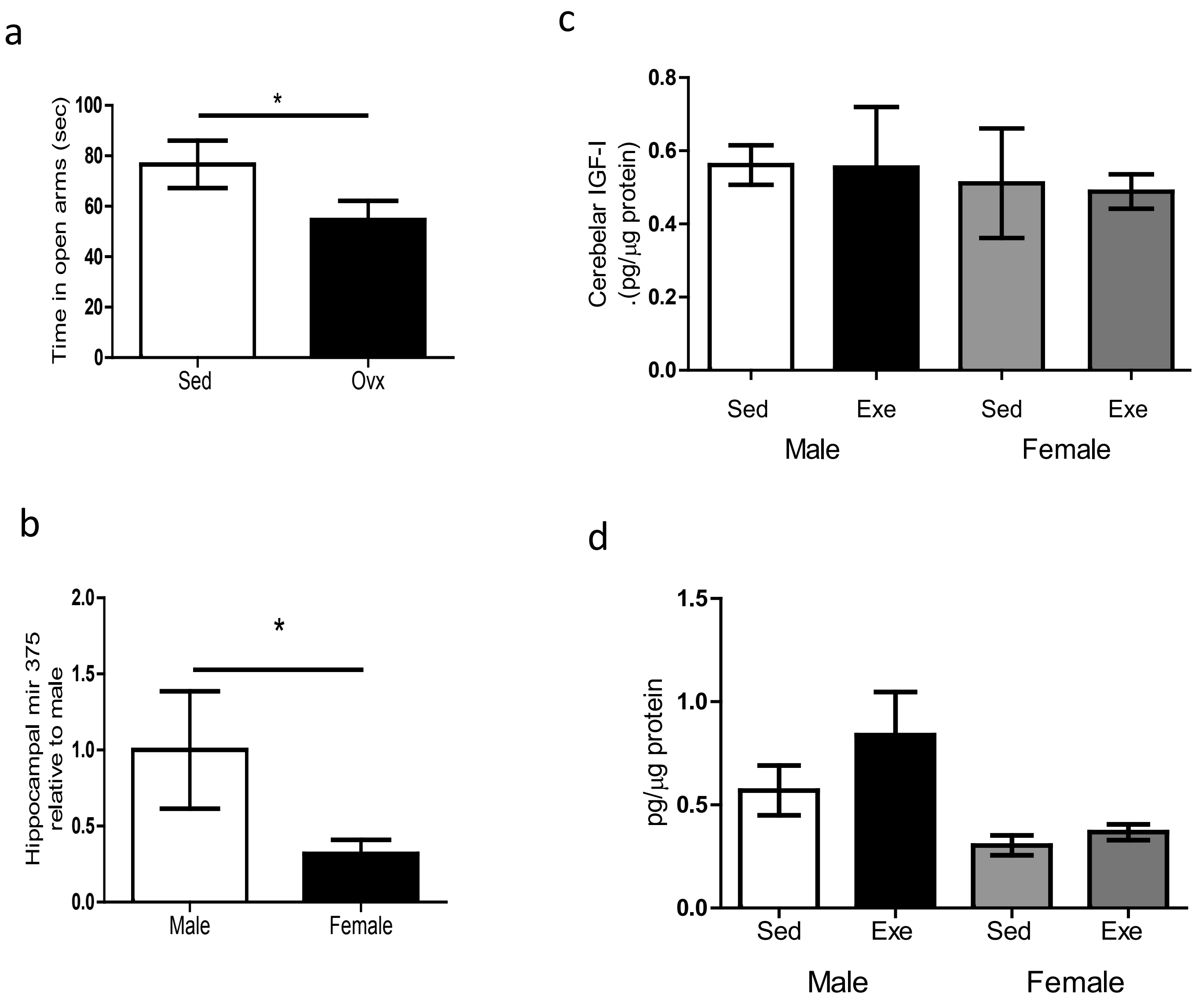

Supplementary Figure 1

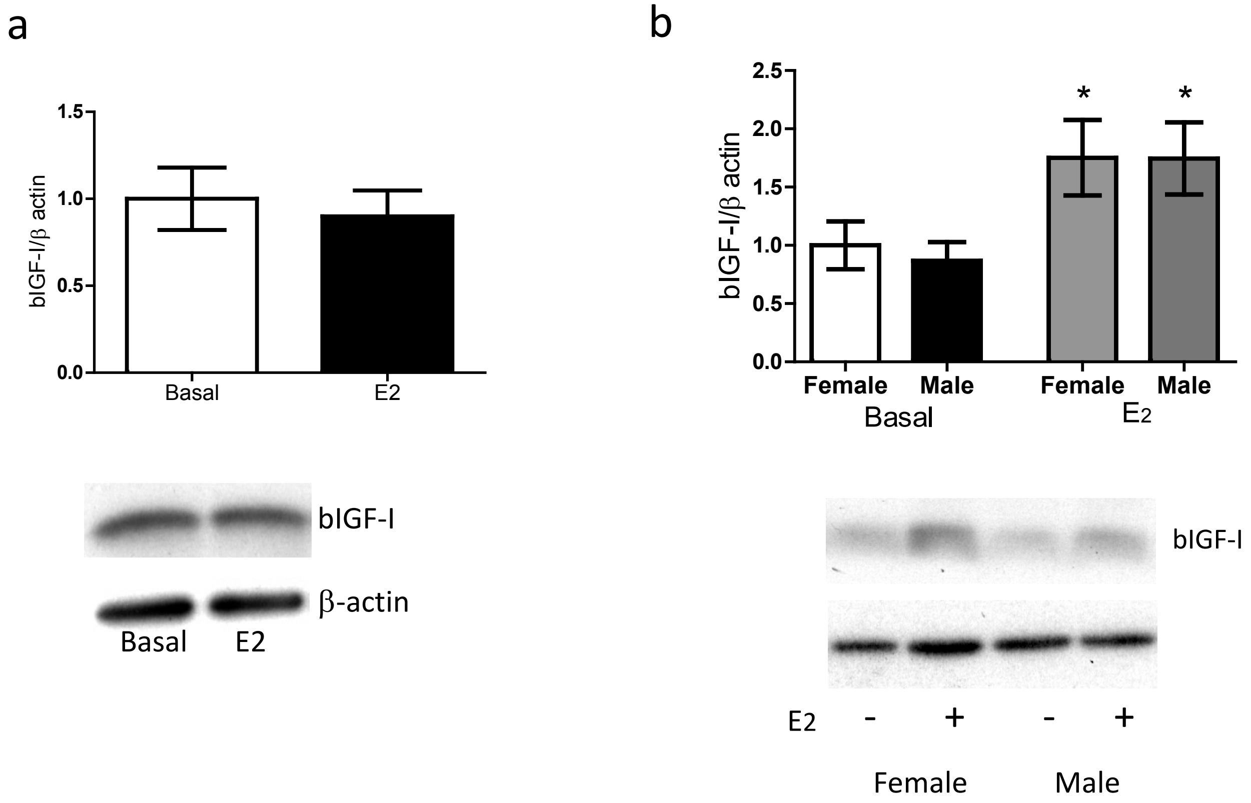

Supplementary Figure 2

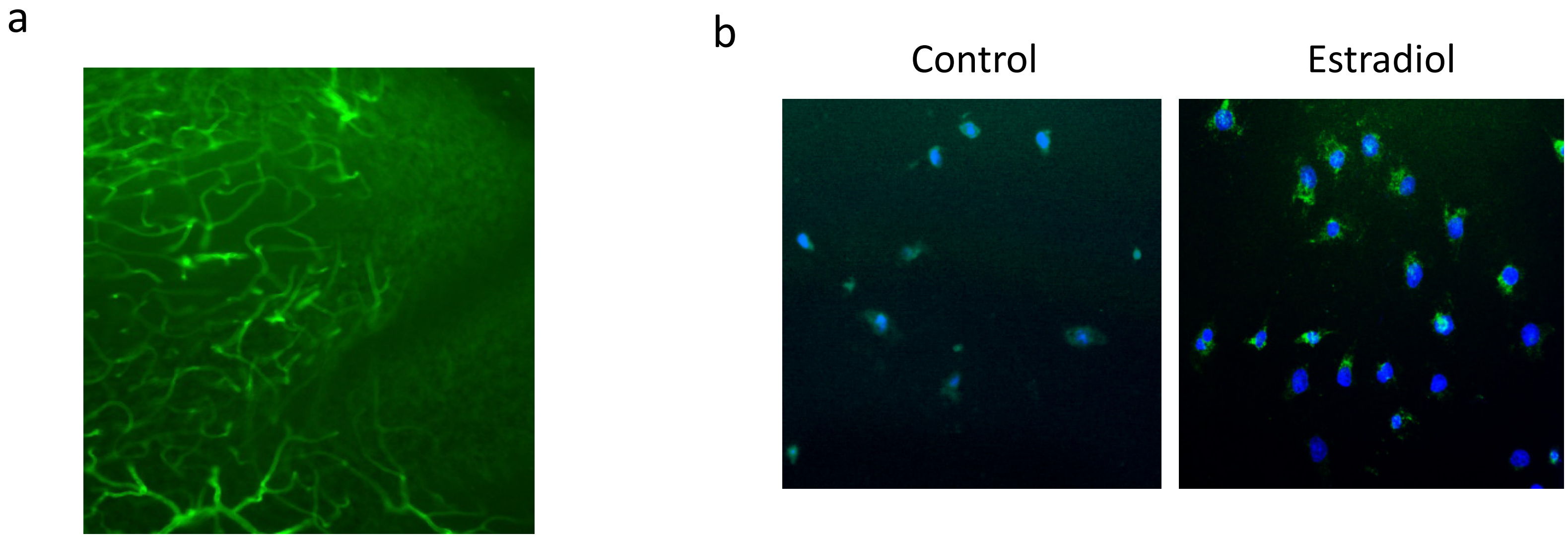

Supplementary Figure 3
